# Psychometric Properties of the Chinese Version of the Brief State Rumination Inventory

**DOI:** 10.3389/fpubh.2022.824744

**Published:** 2022-03-10

**Authors:** Chanyu Wang, Xiaoqi Song, Tatia M. C. Lee, Ruibin Zhang

**Affiliations:** ^1^Laboratory of Cognitive Control and Brain Healthy, Department of Psychology, School of Public Health, Southern Medical University, Guangzhou, China; ^2^State Key Laboratory of Brain and Cognitive Sciences, The University of Hong Kong, Hong Kong, China; ^3^Laboratory of Neuropsychology and Human Neuroscience, The University of Hong Kong, Hong Kong, China; ^4^Department of Psychiatry, Zhujiang Hospital, Southern Medical University, Guangzhou, China

**Keywords:** state rumination, trait-like rumination, reliability, validity, Chinese

## Abstract

State rumination, unlike trait rumination which is described as a persistent and stable response style, is usually triggered by a specific stressful event and causes negative emotions within a short period of time. The measurement methods of trait rumination, such as the ruminative response scale (RRS), are therefore not fully applicable to state rumination. Recently, researchers have developed the brief state rumination inventory (BSRI) to characterize state rumination, addressing the gap in the field of accurate measurement of state rumination. To develop such an effective tool in the Chinese context, we developed a Chinese version of the BSRI and tested its psychometric properties. Two studies were conducted to address the research goal. In Study 1, we recruited 512 subjects, each of whom completed the Chinese version of the BSRI, RRS, emotional regulation questionnaire (ERQ), depression–anxiety–stress scale (DASS), and positive and negative affect scale (PANAS). Results showed that the scores of the BSRI were positively correlated with all other scale scores (*p*s < 0.001), and the correlation with the RRS was the highest, indicating that the BSRI showed good convergent validity. Additionally, the Cronbach's alpha coefficient for the Chinese version of the BSRI was 0.93. Study 2 aimed to examine the ecological validity of the Chinese version of the BSRI. We recruited another 54 subjects who were randomly divided into two groups, with 27 in the rumination induction group and 27 in the distraction group, and recorded the BSRI scores of the two groups before and after a specific experiment. We found there was a significant increase in BSRI scores after rumination induction (*t* = 3.91, *p* < 0.001), while there was no significant difference in the concrete distraction group before and after the experiment (*t* = 0.70, *p* = 0.48). In sum, the Chinese version of the BSRI showed good reliability and validity for assessing state rumination in the general Chinese population.

## Introduction

Rumination is a mode of thinking in which individuals repeatedly ponder over their negative effects or the causes, effects, and results of stressful events they have previously experienced (1). It has been widely reported as an established cognitive vulnerability factor for depression (2). On the one hand, rumination can predict episodes of major depressive disorder (MDD) (3); on the other hand, individuals with MDD usually maintain a high rumination state even if the depressive symptoms are partially or completely alleviated (4). Furthermore, rumination has been proved to postpone the treatment response of MDD and reduce the efficacy of drugs used for the treatment of depression (5). Beyond MDD, other mental disorders such as anxiety disorders, substance abuse, and bulimia nervosa are also affected by rumination (6, 7). Therefore, rumination could be regarded as a “transdiagnostic factor,” a better understanding of which would be of great significance to promote mental health (8, 9).

Early theoretical models of rumination such as Nolen-Hoeksema's Response Styles Theory (10) considered rumination as a relatively stable and chronic trait. Individuals with such a trait-like rumination style were viewed as tending to produce a sustained but unproductive focus of attention on negative outcomes and their associated feelings (11). Trait rumination can usually be assessed by self-rated scales, such as the ruminative response scale (RRS) (12) and the repetitive thinking questionnaire (RTQ) (13). However, simply categorizing rumination as a trait is not convincing. Recent studies have suggested that rumination is also a state-like or a temporary cognitive response, which is highly dependent on situational cues and maybe instantaneously induced when individuals perceive a difference between their current state and their anticipatory goal (14, 15). Therefore, in control theory, Martin et al. referred to temporary maladjustment, which is triggered by specific situational factors (e.g., exposure to acute stressors), as state rumination (16). State rumination, like trait rumination, can also lead to negative outcomes, such as anger (17). The negative effects of state rumination have been verified by empirical studies on laboratory-induced rumination (18–20). Nevertheless, the negative consequences of state rumination on mood and psychological functioning are independent of trait rumination, even though state rumination is transient and dependent on environmental factors (21). For example, when the level of trait rumination is controlled, an increase in state rumination still leads to the reinforcement of negative emotion (22). Additionally, state rumination *per se* is still related to a lower level of recovery from stress even after controlling for trait rumination and depressive symptoms (20). Furthermore, in terms of measurement, the test–retest correlation of RRS between two interviews was 0.67 in Nolen-Hoeksema's study (23). This suggests to some extent that rumination is not a static structure, which further underscores the importance of assessing state rumination (20). Thus, the characteristics of state rumination cannot be completely covered by trait rumination, while trait rumination measures such as the RRS scale are not fully applicable to state rumination.

Due to the difference in concepts, assessment of state rumination differs from that of trait rumination, which can be measured directly and separately. For state rumination to be induced, it usually requires manipulation before the measurement (20). Under laboratory conditions, researchers mainly instruct participants to imagine the content described in specific sentences to induce their rumination and then measure whether rumination has been successfully induced by evaluating changes in emotion (24). Only when the induction process is confirmed to be effective, it is appropriate to assess state rumination. Existing studies typically assess state rumination by asking participants the degree of feelings and problems they are currently concerned with using one or two Likert-type items; however, the limited number of items may lead to ceiling or floor effects (25). Although other researchers attempted to use the state rumination questionnaire (SRQ) to assess state rumination, they also pointed out many limitations of the SRQ, including that the questionnaire has not yet been psychometrically evaluated, and some items may be confused with sadness ratings (20). To this end, Marchetti et al. (26) compiled the brief state rumination inventory (BSRI). The BSRI, a self-report measurement using the visual analog scale (VAS), characterizes the individual's intensity of repetitive and persistent negative thoughts, negative views about self, and the perceived uncontrollability of the current situation. It has been shown to have satisfactory levels of internal consistency in addition to convergent and divergent validity. Moreover, the BSRI also showed sensitivity to detecting changes in the intensity of rumination within a daily life context. Recently, it has been successfully translated into Turkish with good reliability and validity, charting state rumination (27). The BSRI could be an optimal measure, to detect the short-lived increases and decreases in rumination as well as the factors that trigger such fluctuations. However, a Chinese version of this scale has not been developed, which means the measurement method cannot currently be used in Chinese rumination studies. In view of the increasing number of research studies on rumination in China [e.g., (24, 28)], it is necessary to develop a Chinese version of the BSRI and test its reliability and validity in the Chinese population to provide an alternative tool for researchers to study rumination.

Researchers regard rumination as a process of persistently thinking about one's emotions and problems to better resolve and make out the meaning of one's circumstances (29). However, rather than promoting adaptive change, rumination incapacitates individuals and exacerbates symptoms by reinforcing negative thinking and impeding problem-solving (1). It has maladaptive consequences, triggering a more negative affect (NA) (30) and fewer positive emotions (positive affect, PA) (31). Moreover, rumination has been associated with a reduced ability to recognize, monitor, and regulate emotions (32). Thus, many researchers have linked rumination to emotion regulation (ER) (14, 33). For example, trauma-exposed individuals respond to distress resulting from ER difficulties with rumination (33). Moreover, rumination is also related to anxiety and depression (29). Therefore, it is necessary to consider these factors when exploring rumination. Herein, scales such as the depression–anxiety–stress scale (DASS), emotion regulation questionnaire (ERQ), and positive and negative affect scale (PANAS) are used to detect (mal)adaptive effects could be suitable to help examine the criterion validity of rumination scales (34).

The current study aimed to develop a Chinese version of the BSRI and had two subgoals. The first was to test the reliability and validity of the Chinese version of the BSRI, to provide a more accurate and effective vehicle for evaluating state rumination. Therefore, we conducted Study 1, which examined the internal consistency, factor structure, and convergent and concurrent validity, as well as the incremental validity of the BSRI using sufficient samples. The second subgoal was to test whether the Chinese version of the BSRI is applicable for the measurement of state rumination after actual rumination induction. To this end, we conducted a behavior study (Study 2) assessing the BSRI's sensitivity to momentary changes in rumination under a rumination induction context to examine the ecological validity of the Chinese form of the BSRI. We predicted that the reliability and validity of the Chinese version of the BSRI could reach the level of the original BSRI (26).

## Study 1

### Participants

We collected data from 519 subjects through questionnaires. None of the participants had any mental disorder. After removing seven invalid responses due to missing data, the remaining 512 participants (336 females; age, *M* = 22.19, *SD* = 4.60, range: 18–50) were included in our analyses (Table 1). Of the participants, 426 (83%) received college or higher education and 86 (17%) have a high school education or less than. There are 298 (58%) participants living in town and 214 (42%) in the village. Data on only child was 188 (37%) and the rest of them, about 324 (63%), had other siblings. Of note, the sample size (*n* > 500) is very good for questionnaire studies according to the recommendations of previous studies (35).

**Table 1:** Participants characteristics.

	**Study 1**	**Study 2**
Age, years	22.19 (18–50)	21.52 (19–25)
Sex		
Male	176 (34%)	11 (20%)
Female	336 (66%)	43 (80%)
Education		
University	426 (83%)	52 (100%)
Others	86 (17%)	0 (0%)
Homeplace		
Town	298 (58%)	33 (64%)
Village	214 (42%)	19 (37%)
Only child or not		
Yes	188 (37%)	19 (37%)
No	324 (63%)	33 (63%)

### Measures

#### Brief State Rumination Inventory

The Chinese version of the BSRI was translated by two psychology postgraduate students with the permission of the original developer, Marchetti (26). They translated the BSRI items from English to Chinese and then performed a back-translation to further test the translation accuracy. On the basis of guaranteeing the original meaning, we ensured that the compiled items conformed to the characteristics of Chinese expression as much as possible. The Chinese version of the BSRI consists of eight forward-scoring items, and the items are scored on a 100 mm VAS ranging from 0 (“strongly disagree”) to 100 (“strongly agree”). The total score is obtained by summing up all items. According to the original version of the BSRI (26), it has satisfactory levels of internal consistency (α = 0.91) and positive correlations with related constructs such as negative affect, trait rumination, and depressive symptoms.

#### Ruminative Response Scale

We chose the Chinese version of the RRS revised by the Chinese scholars Han and Yang (36), which was translated on the basis of the original scale compiled by Nolen-Hoeksema (1). The RRS consists of 22 items, divided into three dimensions including brooding, reflective pondering, and symptom rumination, and all items are rated on a 4-point scale ranging from 1 (“never”) to 4 (“always”). A higher score indicates a higher level of rumination. In this study, the α coefficient of the scale was 0.94.

#### Depression–Anxiety–Stress Scale

The DASS used in this study was a Chinese version translated by Gong et al. (37) on the basis of the original scale compiled by Lovibond (38). The DASS is divided into three subscales: depression, anxiety, and stress. Each subscale contains seven items rated on a 4-point scale from 0 (“did not apply to me at all”) to 3 (“applied to me very much or most of the time”). Each subscale score is calculated by adding the scores of the seven items it contains. In this study, the α coefficients of the depression, anxiety, and stress subscales were 0.80, 0.87, and 0.85, respectively.

#### Emotion Regulation Questionnaire (ERQ)

The Chinese version of the ERQ was developed by Wang et al. (39) based on the process model of emotion regulation (ER) proposed by Gross and James (40). The ERQ consists of 10 items, with each item being rated on a 7-point scale from 1 (“strongly disagree”) to 7 (“strongly agree”). Moreover, the ERQ can be separated into two dimensions, reappraisal and expressive suppression, in which items 1, 3, 5, 7, 8, and 10 measure reappraisal, and items 2, 4, 6, and 9 measure expressive suppression. The α coefficients of the reappraisal and expressive suppression subscales in this study were 0.76 and 0.82, respectively.

#### Positive and Negative Affect Scale

The Chinese version of the PANAS, including positive emotions (interested, alert, attentive, excited, enthusiastic, inspired, proud, determined, strong, active) and negative emotions (upset, distressed, nervous, jittery, guilty, ashamed, hostile, irritable, scared, afraid), developed by Huang et al. (41), was rated on a 5-point scale from 1 (“not at all”) to 5 (“extremely”). The α coefficients of the positive emotion and negative emotion subscales in this study were 0.87 and 0.88, respectively.

### Procedures

Before the data collection, we obtained permission from the developers of the original form of the BSRI for translation of the scale into Chinese. Next, we invited two psychology postgraduate students to translate the BSRI items from English to Chinese and then performed a back-translation to further test the accuracy of the translation. On the basis of guaranteeing the original meaning, we ensured that the compiled items conformed to the characteristics of Chinese expression as much as possible.

The data collection phase started after we obtained approval from the Institutional Review Board (IRB) of Southern Medical University. Before the presentation of the test items, all participants read the informed consent form and filled out the questionnaires only after they consented to participate in the study. The students who filled the form would receive course credit as a bonus.

### Statistical Analysis

SPSS version 20.0 was used for item analysis, reliability analysis, validity analysis, and exploratory factor analysis (EFA). Additionally, the lavaan package in R was used to conduct a confirmatory factor analysis (CFA).

## Results

### Construct Validity

We analyzed the BSRI data and found that Bartlett's test of sphericity yielded a chi-square value of 2,881.07 (*df* = 28, *p* < 0.001) with a KMO index of 0.90, indicating that the BSRI was suitable for factor analysis. Then, EFA was used to obtain the factor structure of the scale. The common factor was extracted by principal component analysis (PCA), and we finally extracted only one factor according to the principle of eigenvalue >1. The eigenvalue of the extracted factor was 5.23, explaining 65% of the total variance. Factor loadings of each item ranged from 0.78 to 0.84, indicating that all the individual items were meaningful and so should be retained. Item analysis was also performed by conducting an independent two-sample *t* test on the scores of the two groups (the top 27% and bottom 27% participants ranked according to the total BSRI score) for each item of the BSRI, showing that the scores of the high-score group in each item were significantly higher than those of the low-score group (*p* < 0.001).

To test whether the single-factor model fit the Chinese version of the BSRI, CFA was performed using the lavaan package of R software. The results confirmed the EFA-based *a priori* factor structure and the model demonstrated acceptable fit (χ^2^ = 239.64, *df* = 14, *p* < 0.001, CFI = 0.90, TLI = 0.85, RMSEA = 0.058), further supporting the original BSRI factor structure. We further calculated Pearson's correlation coefficients between items and items, item scores, and total scores. There were significant positive correlations among all item scores (*r* = 0.43–0.77, *p* < 0.001), and there were also significant correlations between item scores and total scores (*r* = 0.78–0.84, *p* < 0.001) (Table 2).

**Table 2:** Each item factor load, common factor variance, correlation matrix of items, and total scores in Chinese version of BSRI (*n* = 512).

	**Mean (SD)**	**PCA factor load**	**EFA factor load**	**Item 1**	**Item 2**	**Item 3**	**Item 4**	**Item 5**	**Item 6**	**Item 7**	**Item 8**	**Total**
Item 1	42.37 (31.06)	0.78	0.73	1								
Item 2	43.56 (32.20)	0.79	0.74	0.68^***^	1							
Item 3	42.66 (32.58)	0.83	0.79	0.71^***^	0.77^***^	1						
Item 4	40.78 (32.19)	0.82	0.79	0.56^***^	0.57^***^	0.65^***^	1					
Item 5	46.69 (32.26)	0.80	0.77	0.58^***^	0.54^***^	0.57^***^	0.60^***^	1				
Item 6	49.26 (33.06)	0.83	0.82	0.55^***^	0.55^***^	0.56^***^	0.64^***^	0.66^***^	1			
Item 7	37.05 (31.60)	0.78	0.75	0.49^***^	0.43^***^	0.54^***^	0.64^***^	0.57^***^	0.64^***^	1		
Item 8	46.65 (33.05)	0.84	0.83	0.52^***^	0.57^***^	0.59^***^	0.63^***^	0.65^***^	0.78^***^	0.71^***^	1	
Total	349.02 (208.74)	–	–	0.78^***^	0.79^***^	0.83^***^	0.82^***^	0.80^***^	0.83^***^	0.78^***^	0.84^***^	1

*The correlation coefficient was calculated by Pearson product correlation*.

*** *Significant p < 0.001*.

### Criterion Validity

We chose the RRS, DASS, ERQ, and PANAS as the criterion questionnaires in our study. Correlation analysis results showed that there were significant positive correlations between the total score of the BSRI and each subscale of RRS, each subscale of DASS, the reappraisal subscale of ERQ, and the negative emotion subscale of PANAS (*P*s < 0.001) (Table 3). Moreover, further analysis showed that the correlations between the BSRI and subscales of RRS were significantly higher than that of other scales (such as DASS and PANAS), indicating that the BSRI had good convergent validity.

**Table 3:** Criterion validity analysis.

	**BSRI**	**RRS-S**	**RRS-B**	**RRS-R**	**ERQ-R**	**ERQ-E**	**DASS-D**	**DASS-A**	**DASS-S**	**PANAS-PA**	**PANAS-NA**
BSRI	1										
RRS-S	0.42^***^	1									
RRS-B	0.43^***^	0.81^***^	1								
RRS-R	0.51^***^	0.70^***^	0.74^***^	1							
ERQ-R	0.24^***^	0.20^***^	0.19^***^	0.20^***^	1						
ERQ-E	−0.03	−0.11^*^	0.02	0.03	0.11^*^	1					
DASS-D	0.40^***^	0.71^***^	0.59^***^	0.51^***^	0.09	−0.17^**^	1				
DASS-A	0.32^***^	0.71^***^	0.50^***^	0.45^***^	0.16^***^	−0.24^***^	0.77^***^	1			
DASS-S	0.31^***^	0.64^***^	0.49^***^	0.44^***^	0.15^**^	−0.22^***^	0.78^***^	0.76^***^	1		
PANAS-PA	−0.05	−0.35^***^	−0.18^***^	−0.14^**^	−0.02	0.28^***^	−0.31^***^	−0.46^***^	−0.28^***^	1	
PANAS-NA	0.34^***^	0.66^***^	0.52^***^	0.42^***^	0.13^**^	−0.19^***^	0.68	0.60^***^	0.67^***^	−0.19^***^	1
Mean (SD)	349.01 (208.94)	24.30 (6.5)	11.19 (3.07)	10.56 (2.99)	14.88 (4.92)	31.08 (5.26)	4.19 (3.16)	3.12 (3.05)	3.13 (2.65)	30.54 (6.05)	23.54 (6.47)

*Chinese version of BSRI, the rumination response scale (RRS), the emotion regulation questionnaire (ERQ), the Depression Anxiety Stress Scale (DASS) and the positive and negative affect scale (PANAS) (n = 512). RRS-S, symptom rumination subscale of RRS; RRS-B, brooding subscale of RRS; RRS-R, reflective pondering subscale of RRS. ERQ-R, reappraisal subscale of ERQ; ERQ-E, expressive suppression subscale of ERQ. DASS-D, depression subscale of DASS; DASS-A, anxiety subscale of DASS; DASS-S, stress subscale of DASS. PA, positive effect; NA, negative effect. The correlation coefficient was calculated by Pearson product–moment correlation*.

* *Significant p < 0.05;*

** *Significant p < 0.01;*

*** *Significant p < 0.001*.

### Reliability Analysis

The internal consistency analysis showed that the α coefficient of the BSRI was 0.93. Additionally, the Spearman–Brown split-half reliability of the BSRI was 0.86.

## Discussion

In Study 1, the Chinese version of the BSRI retained all eight items, and each item had high discrimination power. There were significant positive correlations among the items and between scores of items and total scores, indicating that both the items and the whole scale had a high level of homogeneity. Moreover, the EFA results showed that the Chinese version of the BSRI was a single-factor model, which is consistent with the theoretical hypothesis and research results of Marchetti (26), the design of the original questionnaire, indicating that the BSRI has relatively stable cross-cultural consistency.

Validity refers to the degree to which a measuring tool can accurately measure what is being measured. The significant positive correlation between the BSRI and criterion scales (i.e., RRS and DASS) indicates that the Chinese version of the BSRI has good validity. This is consistent with previous studies where state rumination was similarly correlated with trait rumination and depressive symptoms (42). RRS was considered as one of the criterion scales in the calculation of criterion validity because although there are differences in the psychological mechanism and manipulation mode of state rumination and trait rumination (43), they both belong to the concept of rumination in essence. The BSRI was moderately correlated with three RRS subscales but not highly correlated (*P*s < 0.6), which was consistent with the results of prior study, indicating that the BSRI captures state aspects of rumination that are not subsumed under trait rumination (26). Among the three subscales, reflective pondering refers to the tendency to think about symptoms of depression and negative life events with an attitude that leaves space for problem-solving. The correlation between reflective pondering with the BSRI is the highest (*p* = 0.51), which may indicate the state rumination is more correlated with stressors encountered in life. Additionally, it is particularly noteworthy that the correlations between the BSRI and RRS subscales were significantly higher than those between BSRI and other criterion scales, which also indicates that the Chinese version of the BSRI has good ecological validity and can be a valid measure for state rumination.

Reliability reflects the consistency and stability of measurement results under different conditions. The α coefficient of the BSRI in the Chinese version is 0.93. Social science research on the reliability of scales generally considers that a questionnaire has high reliability when its α coefficient is greater than 0.70 (44). Thus, just as the English version of the BSRI developed by Marchetti can be used for group tests, the Chinese version of the BSRI can also be used as a suitable tool for large-scale assessment of state rumination.

## Study 2

Study 1 revealed that the Chinese version of the BSRI had good reliability, criterion validity, and construct validity, indicating that the BSRI had high stability and accuracy for measuring state rumination. However, it is inadequate to judge the extent of its applicability without an assessment of the real-world use of the questionnaire (45). Additionally, recent studies have suggested that scales demonstrating ecological validity may have greater utility in clinical research, as well as in the prevention and intervention efforts (46). Therefore, to further test whether the scale is suitable for actual experiments and to test the ecological validity, we conducted a behavioral experiment in which we induced individuals' state rumination in a laboratory and tested whether the BSRI could sensitively measure the induced effect. We predicted an increase in BSRI scores following the rumination induction but not the control induction and hypothesized that the BSRI had good ecological validity that can sensitively detect the difference between before and after inducing state rumination. Moreover, since rumination contributes to negative mood (1), we deduced participants in the rumination condition would experience more negative effects compared to those in the control condition.

### Participants and Procedures

A total of 60 subjects signed up for our experiment by scanning QR code on the poster posted online and completed the RRS and the depression subscale in the DASS. Only participants who had completed questionnaires and did not show significant depressive symptoms were included in this study. Finally, we included 54 subjects (43 females; age, *M* = 21.52, *SD* = 1.33, range: 19–25) (Table 1). It is important that each group should include at least 17 participants to detect medium effect sizes of 0.25 with a power of 0.80 using the 2 × 2 mixed-design ANOVA. We have recruited 27 participants in rumination and distraction group, separately.

Then, we randomly divided them into an experimental group (rumination group: 27 subjects including 23 females; age: *M* = 21.64, *SD* = 1.39) and a control group (distraction group: 27 subjects including 20 females; age: *M* = 21.00, *SD* = 1.20). In the experimental group, a rumination task was administered, which is a rumination protocol used by previous studies (1, 47–49). Participants in the rumination group were guided by particular sentences (45 items, such as “think about why things developed in this way”), while participants in the control group were asked to think about concrete things (45 items, such as “think about what you ate last night”). All stimuli were presented by E-prime3.0, and each item was presented for 14 s. The BSRI measurement was performed before and after the experiment to measure the state rumination. It is worth noting that rumination induction usually leads to emotional changes. Therefore, positive and negative emotions before and after the experiment were further assessed by VAS, in which three items were used to evaluate positive emotions, such as “at this moment, I feel happy/excited/joyful,” and three items were used to evaluate negative emotions, such as “at the moment, I feel sad/disappointed/low.” These items were measured on a 100-mm VAS, with scores ranging from 0 (“strongly disagree”) to 100 (“strongly agree”). All subjects received a reward of 20 yuan after participating in the experiment.

### Statistical Analysis

All analyses were conducted using SPSS version 20.0. A 2 (group: rumination group *vs*. distraction group) ×2 (time: pre vs. post) mixed-design analysis of variance (ANOVA) was conducted to check the effect of rumination induction on BSRI scores and thus to examine whether the BSRI was sensitive to temporary changes in the rumination induction context. To further get rid of potential internal correlation matters of the same participant, an independent sample *t*-test was performed to compare the changes of BSRI scores (post-score minus pre-score) of the two groups.

## Results

**Figure 1:**
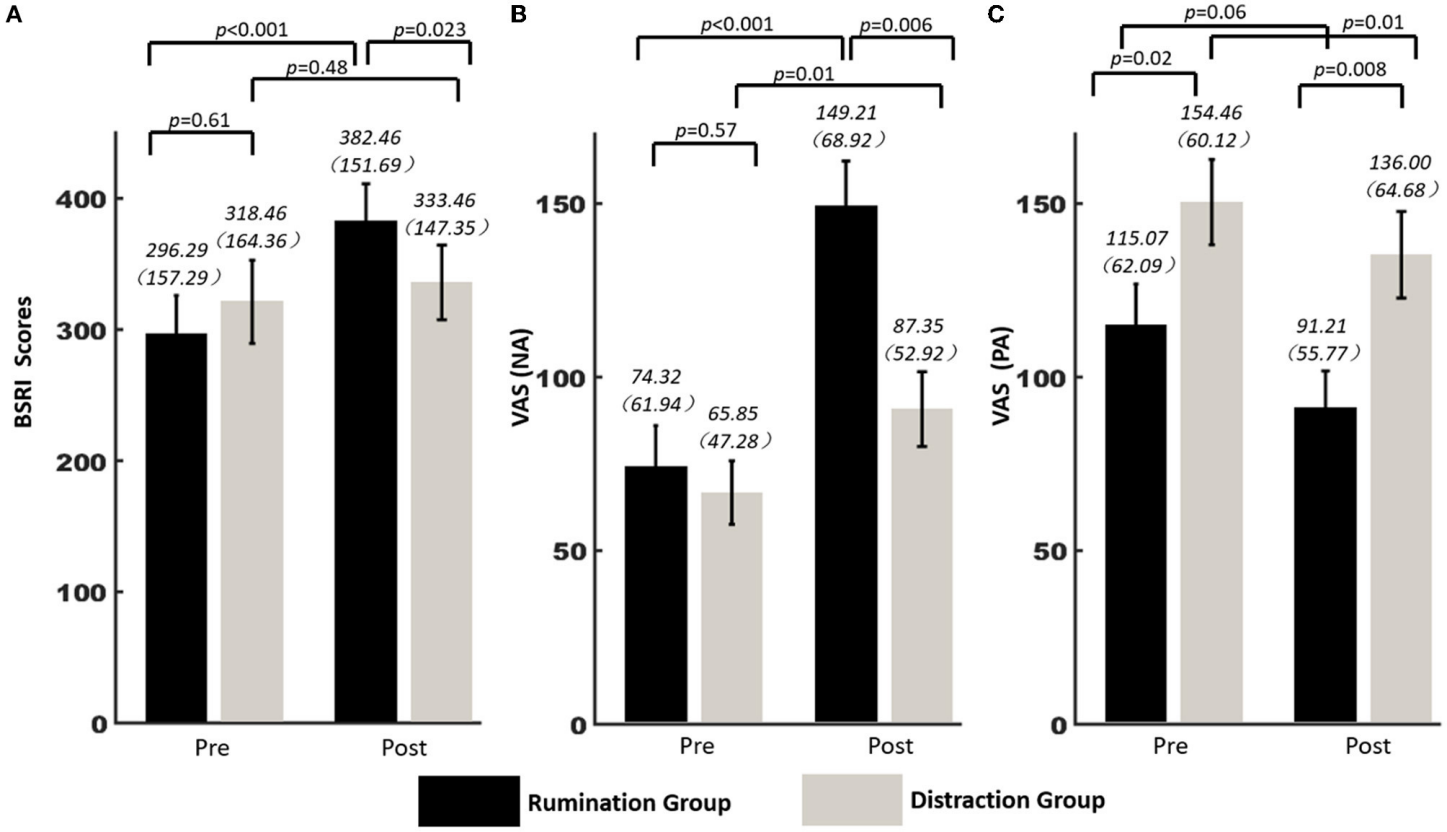
ANOVA results of negative and positive emotion assessment and rumination degree before and after the experiment in Sample 2. **(A)** BSRI scores of rumination group and distraction group before and after the experiment. **(B)** Negative emotion scores of rumination group and distraction group before and after the experiment. **(C)** Positive emotion scores of rumination group and distraction group before and after the experiment. NA, negative affect; PA, negative affect; Pre, before the experiment; Post, after the experiment.

Two independent variables were involved in the data: one was the within-subjects variable “time,” which was divided into preexperiment and postexperiment, and the other was the between-subjects variable “group,” divided into rumination group and distraction group. The 2 × 2 mixed-design ANOVA found a significant main effect of time on BSRI score [*F*_(1, 52)_ = 10.91, *p* = 0.002, η^2^ = 0.173], whereas the main effect of group was not significant [*F*_(1, 52)_ = 0.11, *p* = 0.73, η^2^ = 0.002]. Additionally, it revealed a significant group-by-time interaction [*F*_(1, 52)_ = 5.4, *p* = 0.02, η^2^ = 0.094]. Simple main effects analysis showed that the BSRI score of the rumination group after the induction was significantly higher than that before inducing state rumination (*p* < 0.001), whereas there was no significant difference in the score of the distraction group before and after the experiment (*p* = 0.48) (Figure 1A). Furthermore, the independent samples *t*-test revealed that the changes of BSRI scores (postscore minus prescore) were significantly different between the rumination group and distraction group [*t*_(52)_ = 2.32, *p* = 0.02, *d* = 0.64], which should rule out the potential internal correlation matters of the same participant.

Rumination induction will cause a change of mood, and so the VAS was used to record the positive and negative emotions of the two groups before and after the experiment. A 2 × 2 mixed-design ANOVA was conducted on participants' negative emotion scores. The results showed that the main effect of time [*F*_(1, 52)_ = 48.22, *p* < 0.001, η^2^ = 0.481], the main effect of group [*F*_(1, 52)_ = 5.97, *p* = 0.01, η^2^ = 0.103], and the group-by-time interaction [*F*_(1, 52)_ = 14.79, *p* < 0.001, η^2^ = 0.222] were all significant. Moreover, the posttest results indicated that both the rumination group (*p* < 0.001) and the distraction group (*p* = 0.01) had significantly higher negative emotions after the experiment than before (Figure 1B). For positive emotion, the two-way ANOVA found that both the main effect of time [*F*_(1, 52)_ = 9.06, *p* = 0.004, η^2^ = 0.148] and the main effect of group [*F*_(1, 52)_ = 7.92, *p* = 0.007, η^2^ = 0.132] were significant. The results, however, did not show a significant group-by-time interaction [*F*_(1, 52)_ = 1.47, *p* = 0.70, η^2^ = 0.003] (Figure 1C).

## Discussion

In Study 2, we induced state rumination guided by sentences in one group and compared the BSRI score with that of the control group to test the ecological validity of the Chinese version of the BSRI. We found that the BSRI score of the rumination group after induction was significantly higher than that before inducing state rumination. However, the same effect was not found in the control group. On the one hand, these results showed that the Chinese version of the BSRI can effectively distinguish between the rumination group and distraction group, indicating that it has good ecological validity. On the other hand, it confirms that state rumination is a state-like or a temporary cognitive response, which is highly dependent on situational cues and maybe instantaneously induced when individuals perceive a difference between their current state and anticipatory goal (18–20). It should also be noted that a 2 × 2 mixed-design ANOVA for negative emotion scores showed a significant group-by-time interaction, indicating that the rumination group had a more significant increase in negative emotion after inducing state rumination compared to the control group. This supported the finding from previous studies that rumination is related to negative emotions (29, 50) and provides an empirical basis for exploring the onset and maintenance of depression.

### General Discussion

The purpose of our study was to test the validity and reliability of the Chinese version of the BSRI that we translated from the original text. The main finding of this article is that Study 1 revealed that the reliability of the Chinese version of BSRI was as high as 0.93, and it was positively correlated with many criterion scales measuring trait rumination or negative affect, among which it was most correlated with the RRS. Moreover, Study 1 not only showed good reliability of the Chinese version of the BSRI, but also acceptable construct validity and criterion validity. In Study 2, we revealed the characteristics of effective measurement of state rumination in the Chinese version of the BSRI by inducing state rumination in a subset of participants and comparing the score with another subset of the control group. The results showed that the score of the rumination group increased significantly after inducing state rumination. However, there was little change in the control group. Thus, the Chinese version of BSRI has good ecological validity and is useful for measuring state rumination. All these results suggested that the Chinese version of BSRI provided acceptable psychometric properties and worked well in a Chinese context for assessing state rumination, especially under a rumination induction situation.

It is of great significance to find a valid and reliable measurement to quantify state rumination, especially in China. For instance, accurate and effective measurements of state rumination will provide the basis for cognitive control interventions of rumination. Recent studies have proposed that cognitive control defects are an important reason why individuals fall into rumination (51, 52). Therefore, some researchers in recent years have put forward that cognitive control training can effectively reduce rumination and relieve depressive symptoms (53). However, difficulties emerged relating to assessing state rumination after cognitive control training (54). Due to this, the Chinese version of the BSRI, with its good ecological validity, can provide an effective tool for the timely assessment of rumination after cognitive control training and the demonstration of the causal connection between cognitive control and rumination.

The BSRI Chinese localization is also important to further explore the neural mechanism of rumination. In previous paradigms of exploring the neural mechanism of rumination, sentence-induced rumination has mostly been used, and the main indicators for measuring whether rumination is successfully induced are the degree of self-focus and the degree of sadness (24, 55). Therefore, only two scale items are usually used for measurement and both are Likert-type, which may lead to ceiling or floor effect and has marked limitations for judging whether rumination has been successfully induced. However, the Chinese version of the BSRI adopts VAS, which can improve the effectiveness of measurement (56), to more accurately capture the degree of induced rumination. Furthermore, it can also further explore the relationship between individual neural activity and the degree of induction of rumination through correlation analysis, providing a more sensitive tool for probing individual differences in brain activity during rumination.

It should be noted that it is useful to distinguish state rumination from trait rumination more precisely in Chinese with this tool and further explore the different functions and neural mechanisms of the two styles of rumination. Some researchers have found that state and trait rumination have different effects on stress and other diseases (17, 57–59). For instance, state and trait rumination contribute to depression in different ways (58). Another research study has shown that the interaction of trait and state rumination shapes the HPA-axis response to stress (59). In addition, further investigation of the contribution of the two types of rumination to changes in brain activity will help us to reveal aberrant functional connectivity in depression (60). All these areas of research suggest that state rumination differs greatly from trait rumination; it is therefore necessary to find a good measurement to quantify state rumination.

However, there are still some limitations that should be noted. First, the use of the online-based questionnaire format in Study 1 may differ from the original paper-based format in some aspects. Researchers have suggested that although some wording and layout had to be changed for the online mode, these “faithful migrations” were acceptable (61). Additionally, to minimize the difference with the paper questionnaire and ensure the subjects answered the questionnaire carefully, we arranged the experimenters to supervise the questionnaire filling on the site, and then carried out a strict review of each questionnaire. Second, considering that state rumination was a transient state appearing after a specifically induced manipulation and changing over time, the test–retest reliability of the BSRI was not measured in this study. In the future, it may be necessary to seek other indicators to reflect the stability of the scale in this aspect. The persistence of state rumination induced by specific induction methods is also a topic worthy of further exploration. Third, the Study 2 sample mainly comprised of university students. University students are ill-equipped to adaptively manage the sudden stress of emergencies (62), and rumination can be a risk factor for depression among them (63). This group is somewhat representative; nevertheless, future testing needs to further examine the psychometric properties of the questionnaire in a wider population to test its applicability. Fourth, we conducted a behavioral experiment to induce state rumination to explore the ecological validity of the Chinese version of the BSRI, and the results showed good ecological validity. Nevertheless, a growing number of studies have begun to explore the underlying neural mechanisms of rumination. For instance, Vanderhasselt et al. (64) reported that while inhibiting responses to negative information, individuals with high-level rumination showed higher activation than health controls in the fronto–parietal network (FPN), which was the dominant network of cognitive control. Chen et al. (65) also found that in the rumination state, the default mode network (DMN, rumination-related network) and FPN showed the opposite stability of dynamic functional architecture. Hence, it is necessary to combine the Chinese version of BSRI with fMRI study of state rumination in the future.

In sum, this study tested the psychometric properties of the BSRI by recruiting a large number of subjects and examined its ecological validity by laboratory-induced state rumination. The current results of this study indicated that the eight-item Chinese version of the BSRI has good reliability and validity, and it was found to be a useful instrument to measure the level of state rumination in a large-scale population. This is of great significance for the subsequent exploration of the psychopathological and neural mechanisms of rumination.

## Data Availability Statement

The original contributions presented in the study are included in the article/supplementary material, further inquiries can be directed to the corresponding author.

## Ethics Statement

The studies involving human participants were reviewed and approved by the Institutional Review Board (IRB) of Southern Medical University. The patients/participants provided their written informed consent to participate in this study. Written informed consent was obtained from the individual(s) for the publication of any potentially identifiable images or data included in this article.

## Author Contributions

RZ and TL designed the research. CW, XS, and RZ performed the research and analyzed the data. CW and XS wrote the paper. All authors contributed to the article and approved the submitted version.

## Funding

This study was supported by Nature Science Foundation of China (Ref: 31900806). The funding organization played no further role in study design, data collection, analysis and interpretation, and paper writing.

## Conflict of Interest

The authors declare that the research was conducted in the absence of any commercial or financial relationships that could be construed as a potential conflict of interest.

## Publisher's Note

All claims expressed in this article are solely those of the authors and do not necessarily represent those of their affiliated organizations, or those of the publisher, the editors and the reviewers. Any product that may be evaluated in this article, or claim that may be made by its manufacturer, is not guaranteed or endorsed by the publisher.
